# Buckwheat and Amaranth as Raw Materials for Brewing, a Review

**DOI:** 10.3390/plants11060756

**Published:** 2022-03-12

**Authors:** Adriana Dabija, Marius Eduard Ciocan, Ancuța Chetrariu, Georgiana Gabriela Codină

**Affiliations:** Faculty of Food Engineering, Stefan cel Mare University of Suceava, 720229 Suceava, Romania; adriana.dabija@fia.usv.ro (A.D.); marius_ec@yahoo.com (M.E.C.); ancuta.chetrariu@fia.usv.ro (A.C.)

**Keywords:** unconventional raw material, pseudocereals, substitute’s malts, specialty beers

## Abstract

Globally, beer is considered the most-consumed low-alcohol beverage, it ranks third, after water and tea, in the top sales of these drinks. New types of beer are the result of the influence of several factors, including innovations in science and technology, changing requirements for food consumption of the population, competition between producers, promotion of food for health, flavor, and quality, the limited nature of traditional food resource raw materials, and the interest of producers in reducing production costs. Manufacturers are looking for new solutions for obtaining products that meet the requirements of consumers, authentic products of superior quality, with distinctive taste and aroma. This review proposes the use of two pseudocereals as raw materials in the manufacture of beer: buckwheat and amaranth, focusing on the characteristics that recommend them in this regard. Due to their functional and nutraceutical properties, these pseudocereals can improve the quality of beer—a finished product. Additionally, all types of beer obtained from these pseudocereals are recommended for diets with particular nutritional requirements, especially gluten-free diets. Researchers and producers will continue to improve and optimize the sensory and technological properties of the new types of beer obtained from these pseudocereals.

## 1. Introduction

Beer is the most widely consumed low-alcohol beverage, and the annual per capita consumption, which is estimated to be 5.11 million hL/day, is increasing year by year [1]. Consumer interest in this beverage has increased due to unprecedented assortment diversification and the reinvention of craft beer [1,2]. Over the last 40 years, as scientific discoveries have continued to develop, amazing innovations have resulted in advances in the quality of the finished beer product. Innovation in the beer industry often involves the use of new mixtures of cereals and pseudocereals or the rediscovery of old cereals, new hop varieties or hop substitutes, new non-*Saccharomyces* yeast crops, fruit, vegetables, some spices, and other flavouring compounds to improve/modify the sensory characteristics of the finished product, customize a type of beer or offer a new type of different beer [3,4,5]. However, minimal progress has been made in considering raw ingredients, without malting, in the brewing process [6]. Moreover, there are few data in the literature on the use of unconventional raw materials in brewing recipes and on how they influence the physico-chemical and sensory characteristics of the finished product [7].

**Figure 1:**
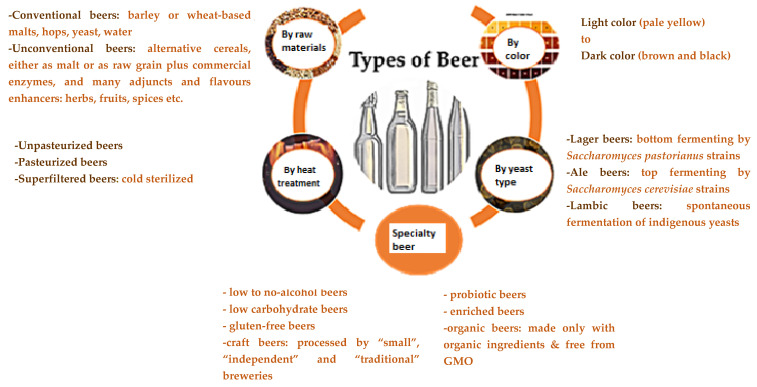
Types of beer according to different classification criteria.

Conventional raw materials in beer processing include barley or wheat malt, water, hops, and yeast, to which unmalted cereals and possibly enzymatic preparations can be added in various proportions, through a technological process that includes four main operations: malting, mashing/filtration, boiling/hopping and wort pitching/fermentation [8,9,10]. Assortment diversification has been influenced by several factors, such as the rebirth of craft beer; increased consumer interest in functional beer; the concern of producers to reduce the costs of obtaining the finished product; development of gluten-free brewing; consumer demand for the unique experience of consuming authentic products of superior quality, with distinctive taste and aroma; and unfavourable conditions for growing barley or wheat in some parts of the world [8,11,12,13]. These emerging trends and new developments in the beer market have led to the production of distinctive and unconventional products, which, together with traditional beers, make up many beer types (Figure 1).

Producers’ interest in making a non-traditional beer requires technical and empirical knowledge of the composition of the new ingredients in the production recipe, and knowledge of the variables of each stage of the technological process. For example, craft beer is a type of beer in which “anything is possible” in the world. Medicinal plants, herbs, fruits, and spices are a treasure trove of bioactive components, making them valuable antioxidant raw materials for beer [14]. The growing interest in craft beer has contributed to an increase in the number of microbreweries, which in 2017 accounted for 94% of the over 19,000 breweries worldwide [4]. 

The distribution of craft beer producers worldwide is as follows: the United States and Europe hold 46% and 43%, respectively, followed by Canada (4.5%), South Africa (4.5%), Australia (3%), Japan (1.6%) and New Zealand (1%). In 2019, the United States ranked 1st in the world in the number of small breweries (8386, with over 20,000 brands of craft beer), with the craft beer market accounting for 13.6% of the market share [3].

Craft beer brewers develop their beer with imagination and creativity and produce many different styles and amazing beers. Innovations in science and technology and the promotion of food for health, flavour, and quality are the engines of the development of new types of beer [15,16,17,18].

The diversification of ingredients in beer production is also imposed by food allergies and intolerances, developing functional beers that aim to combine moderate beer consumption with health benefits [7]. For example, coeliac disease is an incurable disease, and the only therapy is a strict, rigorous, gluten-free diet throughout life [19]. According to Codex Alimentarius and EU Regulation 41/2009 for gluten-free foods, beers with less than 20 mg/kg of gluten can be declared as gluten-free beers [20]. Barley (*Hordeum vulgare*), wheats (*Triticum aestivum*, *Triticum turgidum ssp. durum*, *Triticum turgidum ssp. turgidum*, *Triticum turanicum*, hulled wheat: *Triticum monococcum*, *Triticum dicoccum*, *Triticum spelta*), rye (*Secale cereale*), and oats (*Avena sativa,* *triticale (x Triticosecale)* and *tritordeum*), respectively, can trigger coeliac disease [9,21,22,23].

In recent years, studies investigating the use of 100% alternative ingredients instead of barley malt in gluten-free beer production have increased [24]. Raw materials that have been investigated for making gluten-free beer include cereals such as sorghum, maize, rice, and millet, and pseudocereals such as buckwheat, amaranth, and quinoa [24,25,26,27]. However, techniques for producing beer from cereals (other than barley and wheat) and pseudocereals are not yet well developed. A crucial element in gluten-free beers is their taste, which for pseudocereals differs significantly from the taste of traditional beers [8].

Pseudocereals do not belong to the *Poaceae* family, which includes barley and wheats, so they do not contain gluten-generating proteins and can be used to make gluten-free beer [24]. Amaranth (*Amaranthus* spp.), buckwheat (*Fagopyrum esculentum*), and quinoa (*Chenopodium quinoa*), due to their high starch content, are recommended to be used in obtaining value-added foods [24,28,29,30]. Pseudocereal malt is characterized by a high content of protein, carbohydrates, fibres, minerals, and vitamins. For example, in Poland, buckwheat, due to its high availability as well as its positive recognition among potential consumers, is used to obtain buckwheat malt, but its quality does not allow obtaining 100% buckwheat malt beer without enzymes addition [31]. Buckwheat is generally one of the most cited pseudocereals in the literature for the manufacture of gluten-free malts and beers, as it has shown excellent results over the years in terms of productivity and chemical composition of the finished product [27]. Buckwheat is also an important source of antioxidant compounds and its use in brewing considerably increases the antioxidant activity of the finished product. Constant consumption of buckwheat can prevent some “diseases of the civilization born of food” (indigestion, obesity, constipation, cholesterol, diabetes, hypertension, etc.) [25]. Buckwheat is currently attracting increasing interest as a raw material for functional foods and pharmaceuticals [1]. Due to its excellent nutritional value and the fact that it does not form gluten, buckwheat can be included in gluten-free diets for patients with gluten intolerance. Buckwheat is considered by specialists, due to its huge nutraceutical properties, to be the “golden culture” of the future [2,26].

Amaranth is a plant that has been a staple for about 8000 years. In America, it was considered a sacred food for the Inca, Mayan, and mainly Aztec civilizations due to its nutritional and therapeutic properties and ritual uses [32]. Today, it is an underused, rediscovered crop that includes species that are grown as leafy vegetables, grains, or ornamental plants, while others are weeds. Amaranth is one of those rare plants whose leaves are eaten as a vegetable, while the seeds are used as cereals, with a multipurpose potential that is worth exploring [33,34,35].

Buckwheat and amaranth are two pseudocereals that have multiple uses, including for obtaining of malt and beer, that are grown in different parts of the world. This review treats these two pseudocereals as possible substitutes for barley malt or wheat malt in brewing. Although obtaining an assortment of beer from ingredients other than barley or wheat is simple, achieving efficiency in the economic process and producing a product acceptable to consumers is an ongoing research challenge.

## 2. Buckwheat and Amaranth: Raw Materials for Brewing

In the process of obtaining beer, the main raw materials currently used for malting are barley and wheat [36]. The many advantages of using these cereals for brewing are well known, one of which is the high starch/protein ratio [37]. The global trend is to replace barley malt or wheat malt with other unconventional raw materials. This is due to consumers’ desire to add new qualities to the finished product, to improve the brewing process, or to reduce the cost of production [38]. This review will detail two pseudocereals, buckwheat and amaranth, which are currently used in brewing, emphasizing the characteristics that recommend them in this regard.

### 2.1. Overview

**Figure 2:**
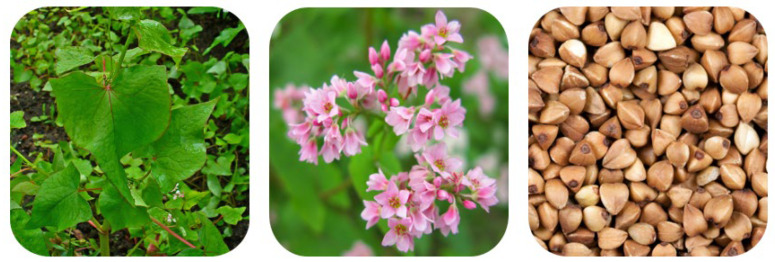
Plant, flowers, and seeds of buckwheat.

Buckwheat is an annual crop with a short development cycle, from 30 to 90 days, which is part of the *Polygonaceae* family, the *Fagopyrum* genus comprising 30 species [39,40,41]. Buckwheat has similar characteristics to cereals such as barley or wheat in terms of chemical composition and edibility, but as it does not belong to the family *Poaceae* is a pseudocereal with differences in grain structure [27,42]. Barley and wheat are monocotyledonous plants, while buckwheat is a dicotyledonous plant. This implies that the triangular buckwheat fruits, similar to those of the beech, have the embryo with two S-shaped cotyledons instead of a cotyledon, and the core reserve compounds are located differently (Figure 2) [42,43,44]. Buckwheat is not related to wheat, and its name is probably related to its triangular seeds and the fact that it has similar uses to wheat [45].

Buckwheat has been grown for centuries for its grains, but also its leaves. However, its cultivation has been neglected during the 21st century due to the increased focus on the development of high-yielding varieties of other cereals, such as rice, wheat, and maize, which has led to a significant decrease in the cultivated area [40,41]. Global buckwheat production has steadily increased, reaching about 4 million tons by 2021 [46]. Buckwheat is widely grown, especially in the northern hemisphere, Asia, Europe and America, China, India, Korea, Bhutan, Nepal, Kazakhstan, Tajikistan, Russia, Ukraine, Lithuania, Estonia, Belarus, Moldova, Poland, Serbia, Croatia, Slovenia, Austria, Italy, United States of America, and Canada [39,47,48,49].

The buckwheat plant is easily adaptable, so that it can be grown almost anywhere in the world and different habitats, from high altitude regions, with low rainfall and temperatures, even in nutrient-poor soils and has a higher resistance to pests in compared to other cereals [43,50,51].

Of the buckwheat species, the common buckwheat (*Fagopyrum esculentum*) and the tartar buckwheat (*Fagopyrum tataricum*) are the most cultivated and consumed species worldwide [39]. From an economic point of view, the most important species is the common buckwheat (*Fagopyrum esculentum*), which represents 90% of the world’s buckwheat production, which is generally grown in the temperate regions of the northern hemisphere [52]. Tartar buckwheat (*Fagopyrum tataricum*) has been called “bitter buckwheat” due to the bitter substances in the grains and has many advantages over other species, such as self-pollination, high grain yield, has better resistance to adverse climatic conditions, being mainly a high-altitude culture [41]. Additionally, tartar buckwheat is considered a healthy food due to the fact that it contains a higher amount of rutin compared to the common buckwheat [53]. There are also wild buckwheat species, the best known being the species *Fagopyrum cymosum*, used, for example, in traditional Chinese human and veterinary medicines. Wild buckwheat species are used by researchers to create newly cultivated species, like in the case of *Fagopyrum giganteum Krotov* species, which was originally defined by Krotov and Dranenko at the Ustymivska Experimental Station in Ukraine, obtained by intraspecific crossings between tartar buckwheat and wild *Fagopyrum homotropicum* [54].

The health benefits of buckwheat have been studied and recognized worldwide. In China, for example, it is said that “people who love buckwheat live a long time” and that “people who love buckwheat are healthy” [55]. The consumption of products containing buckwheat have a hypoglycaemic, hepatoprotective, anticancer, antihemorrhagic, anti-inflammatory, antioxidant, vasoprotective, antihypertensive, and cytoprotective effect, reduces the total triglycerides and total cholesterol in serum and liver, blood sugar, and blood pressure, prevents cardiovascular disease gallstones and cognitive impairments such as Alzheimer’s disease [40,41,54,56]. It can also lead to weight loss as well as a lower risk of diabetes, stroke, and coronary heart disease [51,57]. Soluble and insoluble dietary fibre in buckwheat grains positively affects constipation and obesity [58]. Literature data have shown that long-term consumption of buckwheat products can prevent and control many chronic diseases, such as hyperglycaemia, hypertension and hyperlipidaemia [46].

**Figure 3:**
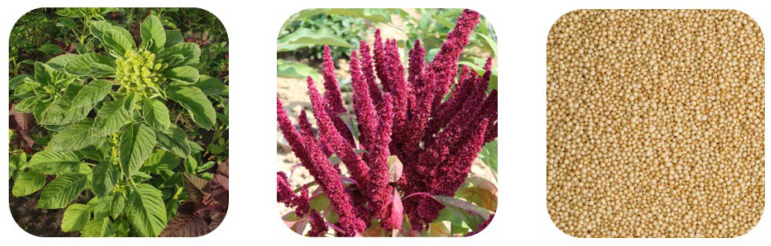
Plant, flowers, and seeds of amaranth.

Amaranth belongs to the order *Caryophyllales*, family *Amaranthaceae*, subfamily *Amaranthoideae*, genus *Amaranthus*, with about 65–70 species, divided into four classes (cereals, vegetables, ornamentals and weeds) which are naturally found in temperate and tropical regions worldwide, mainly on the American continent, at altitudes that vary between sea level and over 3000 m [32,59,60,61,62]. Like buckwheat, it is a pseudocereal, and an annual dicotyledonous plant, with most species originating from Central and South America (Figure 3) [59,63]. Amaranth is currently widely grown all over the world, but mainly in Canada, Mexico, Russia, India, Nepal, China, Indonesia, Malaysia, the Philippines, Kenya, Argentina, Peru, and Australia [35,64]. The world’s largest producer is China [64,65]. Presently, the main consumer market for amaranth seeds is Germany [64].

More than 500 g of seeds can be harvested from a single plant, which can contain 60,000–100,000 pieces [62]. Amaranth is a plant that adapts very well to soil and climate conditions, has a better resistance to biotic and abiotic stress than conventional cereal crops [32,59,66,67]. Due to these advantages, amaranth may be a suitable alternative for cereals that are less tolerant of heat, high radiation, pests, and drought [60,63,64,68].

The name amaranth comes from an ancient Greek word meaning “immortal”, “everlasting”, or “not withering” [35,60].

Among the many species of amaranth discovered, the main typical species grown for seeds are *Amaranthus cruentus*, *Amaranthus caudatus* and *Amaranthus hypochondriacus*, while the most cultivated species for leaves is *Amaranthus tricolor* [59].

The chemical composition and high nutritional value, as well as its great potential for use, have led to the recognition of amaranth by UN/FAO nutrition experts as the plant of the 21st century [59].

The amaranth plant can reach over 2.0 m in height; it has a pivoting root that ensures its survival during periods of water scarcity [32].

In addition to its nutritional value, amaranth has many health benefits. Studies have shown that regular consumption of amaranth has hypocholesterolaemic, antioxidant, antidiabetic, anti-inflammatory, antirheumatic, analgesic, antimalarial, antiemetic, laxative, improves appetite, is antileprotic, induces the decrease of free fatty acids, benefits people hypertension and cardiovascular disease, improves liver function and prevents cancer [32,35,61,69,70,71]. The amaranth’s ability to provide health benefits is due to its bioactive compounds; for example, there is evidence that rutin slows down the aging process, quercetin prevents oxidation, and nictoflorin helps protect memory functions. In some countries, parts of the amaranth plant are used to treat various disorders in traditional medicine. In Zimbabwe, the consumption of amaranth grains has been reported to lead to significant improvements in children’s health, such as improved appetite, quick healing of mouth sores and weight loss. In Benin, the leaves of amaranth are recommended for young children, nursing mothers and patients with constipation, fever, bleeding, anaemia, or kidney problems. In Senegal, the roots are boiled with honey as a laxative for infants. In Ghana, water from macerated plants is used as a wash to treat limb pain. In Sudan and Gabon, the ash from the stems is used as a wound dressing. In Gabon, heated amaranth leaves are used on tumours [35,64].

### 2.2. Structure and Chemical Composition

Specialists’ attention has been directed towards the chemical composition of buckwheat seeds, confirming the unique nutritional value conferred by the various nutritional and bioactive components it contains [39]. The chemical composition is influenced by several factors, such as environmental conditions (especially climatic conditions), growing season, species and variety of origin, crop management practices, and so on [48].

Buckwheat seeds are made up of endosperm where starch is located (on average 70%) and a starch-free aleuronic layer [2,42]. The weight of a thousand grains varies between 17.6 and 25.9 g in commercially available buckwheat seeds, a weight that is directly proportional to the starch content, the useful component for brewing [68]. Buckwheat starch granules are irregularly shaped, in a compact package, measuring 2–6 μm, and contain 24% amylose and 76% amylopectin, which is the usual ratio of cereal starch [43].

The availability of starch varies between 70–91%, an important aspect for the use of buckwheat as a raw material in the beer industry. Starch is also a useful substance that will be the content of fermentable carbohydrates in beer wort [8]. 33% of starch is in the form of resistant starch, which recommends buckwheat as a potential ingredient for the formulation of foods with low glycaemic index [26,72]. Buckwheat contains large amounts of soluble and insoluble dietary fibre, which have a positive effect on constipation and obesity [52].

Buckwheat is recognized as a good source of high biological value proteins that do not form gluten and a balanced amino acid composition (high levels of lysine and arginine, compared to cereals), lipids, antioxidants, organic acids, dietary fibre, mineral substances, and vitamins [2,25,41,42,73].

The protein content of buckwheat is 11–19%, 55% of which is in the embryo and 35% in the endosperm, while the rest is in the shell. Compared to buckwheat, cereal proteins are 10–20% found in the embryo and 80–90% in the endosperm [43]. Buckwheat proteins are easily digested by the human body and are more valuable compared to cereal proteins and by their nutritional value is not inferior to legume proteins [73]. The main protein in buckwheat is a 13S globulin considered a rare vegetable protein with a blood cholesterol lowering effect. Buckwheat also contains lectins with a role in reducing the proliferation of spontaneous and induced tumours [40]. Interestingly, buckwheat protein has a higher amino acid score of 100 compared to cereals [48].

Buckwheat also contains essential fatty acids that are not synthesized in the human body and must come from our diet. The lipid content of buckwheat (0.75–7.4%) is higher than that of wheat, being characterized by a high degree of unsaturation, which is preferable from a nutritional point of view [74]. Buckwheat lipids are resistant to oxidation, which means that buckwheat and processed products, can be stored for a long time [73]. It is also valuable for its phospholipids content, especially lecithin [73,75].

Several biofunctional compounds have also been identified in buckwheat, such as: phenolic acids, phytosterols, bioactive inositols (D-chyrosinitol and myo-inositol), condensed tannins, flavonoids, such as rutin, orientin, homoorientin, vitexin, quercetin, isovitexin and isoorientin [49,56,76,77,78]. *Fagopyrum tataricum* has been found to taste much bitterer than *Fagopyrum esculentum* due to its higher content of phenolic compounds and flavonoids, such as rutin and quercetin [53,57]. Buckwheat also contains phagopyrins and phagopyritol with a huge potential in glycaemic control in people with type II diabetes [40,49,55,79]. Buckwheat rutin is a bioflavonoid with important physiological and biological properties, for example, it keeps capillaries and arteries strong and flexible, in addition to acting as a shield against gastric damage, prevents bleeding, improves vision and hearing, protects against UV light, X-rays, oxidative stress [55,72,80,81]. It should be noted that rutin is not found in any cereals and pseudocereals, so buckwheat can be used as a good source of dietary rutin [2,76].

Buckwheat grains are an important source of minerals (2.0–2.5%), namely zinc, copper, manganese, selenium, phosphorus, potassium, sodium, calcium, iron and magnesium [39,78]. The literature shows differences in the amount of mineral substances of the two cultivated species *Fagopyrum esculentum* and *Fagopyrum tataricum*. While *Fagopyrum tataricum* contains higher concentrations of sulfur, calcium, copper, and molybdenum, *Fagopyrum esculentum* has higher concentrations of selenium, zinc, iron, cobalt and nickel [48]. Buckwheat contains several B vitamins, B_1_ (3.3 mg/kg), B_2_ (10.6 mg/kg), B_3_ (18.0 mg/kg), B_5_ (11.0 mg/kg), B_6_ (1.5 mg/kg), vitamins A, C and E [40,47,78,82].

Amaranth grains, although quite small compared to other cereals, have been widely studied, and there is currently a large volume of literature on the chemical content and nutritional qualities of amaranth [59,60].

Amaranth seeds have a diameter of 0.9–1.7 mm, lenticular in shape, with a weight of 1000 seeds of 0.6–1.3 g [33,59,60,69,83]. The weight of 1000 seeds is an important index for the beer malt industry, as their size is directly proportional to the starch and protein content [59,60,84].

As in the case of cereals, the chemical composition of amaranth seeds is dominated by carbohydrates, of which starch is found in a proportion of 50–60% in relation to the total mass of the seeds or over 90% in relation to the carbohydrates included in the seeds. Starch granules, located mainly in the endosperm, are polygonal in shape and have a high swelling power [59,85]. The amylose content is lower than that of other cereal starches, with values between 0.1% and 11.1%. For this reason, due to the small size of the starch granule, it has specific properties, such as higher solubility, high water binding capacity and susceptibility to enzymes, higher sorption capacity at high water activity range [61]. Amaranth seeds contain higher quantities of dietary fibre (4–8%) than those found in most cereals (2%) [32,86,87].

Amaranth seed proteins are nutritionally valuable, with a high digestibility of about 90%, being rich in essential amino acids, in a proportion close to that recommended by the World Health Organization [60,88,89]. Amaranth grains contain three major proteins, namely, albumin (40%), globulin (20%) and glutelin (25–30%), and only 2–3% prolamine [69]. They do not generate gluten, so amaranth flour is recommended in the diet of people suffering from coeliac disease. Amaranth grains contain lysine, the limiting amino acid in cereals (wheat, rye and triticale), in the amount of 363–421 mg/g N, identical to that contained in soy. Amaranth proteins also contain a considerable amount of amino acids with sulphur (2–5%), methionine, cystine and cysteine, higher than in basic legumes (1.4% on average), such as peas, beans and soybeans [32,59,60,64,85].

Amaranth grains contain 2–3 times more lipids than wheat or rye grains, almost twice as much as maize grains, and contain as many lipids as oat grains [59,60]. Unsaturated fatty acids represent about 76%, the largest share of total fatty acids (unsaturated and saturated), being held by linoleic acid (25–62%), oleic acid (19–35%), palmitic acid (12–25%), stearic acid (2–8.6%) and linolenic acid (0.3–2.2%) [83]. Amaranth oil has been reported as the richest source of squalene in the plant world (with amounts of 7–11% in refined oil) [60]. Squalene has a high content of antioxidants, which prevents oxidative damage induced by free radicals, especially on the skin, and helps to renew the protective layer of the skin [32,64,83]. Amaranth oil is also a rich source of tocotrienols, which is very effective in lowering LDL cholesterol and phytosterols with hypocholesterolaemic effects [32,69]. Among these phytosterols are beta-sitosterol (607 μg/100 g) which is 95% of the total phytosterols, campesterol (8.8 μg/100 g) and stigmasterol (5.6 μg/100 g) [32,90].

The excellent antioxidant capacity of amaranth compared to cereals is conferred by phenolic acids (caffeic acid, p-hydroxybenzoic acid, ferulic acid), flavonoids, phytosterols, squalene and bioactive peptides. These substances give amaranth, among other things, antidiabetic, antihypertensive, immunomodulatory, antitumor, and antimicrobial activities [63,90,91]. Amaranth seeds contain polyphenols in the amount of 14.72–14.91 mg/100 g [61,91].

The mineral content of amaranth seeds is about twice as high as in other cereals [61]. They are a rich source of iron (72–174 mg/kg), calcium (1300–2850 mg/kg), sodium (160–480 mg/kg), magnesium (2300–3360 mg/kg) and zinc (36.2–40 mg/kg) [60]. That is why amaranth flour can reduce the deficiency of calcium, magnesium and iron in gluten-free products and in the gluten-free diet, which can be deficient in these minerals [85,92].

Amaranth seeds contain several vitamins, such as riboflavin (0.19–0.23 mg/100 g), ascorbic acid (4.5 mg/100 g), niacin (1.17–1.45) mg/100 g), thiamine (0.07–0.1 mg/100 g), vitamin E, β-carotene [60,61,63].

The chemical composition of the raw materials is particularly important for the beer industry. Table 1 summarizes the physicochemical characteristics of the two pseudocereals and wheat, one of the conventional raw materials for the beer industry.

From the data presented in Table 1, it can be observed that, compared to wheat, which is a conventional raw material for the production of malt or beer, buckwheat has for the carbohydrate content values of 60–84.1%, very close to those of wheat, of 69–85%. The values taken from the literature for the carbohydrate content of amaranth are lower than those for buckwheat, between 48% and 69%. Regarding the protein content, both buckwheat and amaranth have values of 9.5–18.87% and 13.1–18%, respectively, which are close to those of wheat, 8–19.9%.

### 2.3. Use in the Beer Industry

The use of buckwheat and amaranth as adjuvants in brewing has long been known. Recently, research has been intensified on the use of these pseudocereals and in malted form to obtain new varieties of beer [8]. Recent studies show that these pseudocereals are becoming increasingly popular as raw materials for brewing, mainly due to the additional health benefits outlined in Section 2.1 [103,104].

#### 2.3.1. Buckwheat

The first uses of buckwheat in brewing consisted of its use as a raw material for improving the extracted content of beer wort and reducing production costs. This was materialized by the addition of this pseudocereal in the form of flour, groats, or in the extruded form in the mashing stage. On the other hand, the nutritional value and yield are higher than maize and wheat, and buckwheat is also more resistant than barley and wheat to adverse climatic conditions of the crop [73].

Researchers continued to optimize the malting process with the desire to use 100% buckwheat malt to make gluten-free beer. Barley and wheat, the conventional raw materials used in brewing, are known to contain immunoreactive fragments of gluten-generating proteins, hordein, and gliadin, which can cause allergic reactions in people with coeliac disease [76]. Buckwheat is a dicotyledonous plant as opposed to barley or wheat, which are monocotyledonous plants, so with a different location of the reserve compounds in the grain, as presented in Section 2.2. Due to this difference in structure, the production of enzymes and therefore the malting process may differ between buckwheat and barley or wheat [58].

A synthetic definition of the malting process was provided by Wijngaard et al., 2007 [44]: “Malting is the limited germination of a grain or seed under controlled conditions, a complex physiological process whose conditions are dependent on the structure of the grain”. Deželak et al., 2014 [105] showed that buckwheat has adequate properties to replace barley in the process of industrial production of gluten-free beer, of lower fermentation, and the yeast used in the fermentation process can be reused at least 11 times.

Buckwheat malting includes the technological operations specific to the process of obtaining barley malt or wheat malt, namely steeping, germination, and kilning, a process that produces important physiological changes in the grains. Wijngaard et al., 2006 [106] pointed out that these changes depend on the conditions of malting, the enzymes that affect the grains and the structure of the buckwheat grains. Due to structural and compositional differences between barley and buckwheat grains, malting conditions need to be adapted accordingly. The studies undertaken by specialists focused on optimizing the parameters specific to each stage of the malting process [26,107,108,109,110]. Table 2 summarizes the results of these studies published in the literature. The results obtained so far strongly suggest that buckwheat, when optimally malted, has the potential as a gluten-free alternative to malt for brewing purposes [45,111,112,113,114].

Wijngaard et al., 2005 [106] suggested an optimal buckwheat steeping time in the range of 7–13 h, an interval in which a balance was found between the desired quality and the yield of obtaining the finished malt product. At the end of the steeping operation, the optimum moisture content of the buckwheat varied between 40 and 45%. To optimize germination parameters, Wijngaard et al., 2005 [106] germinated buckwheat at four different germination temperatures, 9.5, 14.9, 16.5, and 20.2 °C, and concluded that the optimal characteristics of the resulting buckwheat malt were obtained when the buckwheat was germinated at 16.5 °C and 20.2 °C. However, they showed that the amylolytic activity was lower compared to that of barley malt, a suggested solution to remedy this was to prolong the malting time. Compared to barley malt, buckwheat malt also had a low level of fermentable carbohydrates and a high friability [44].

One year later, Phiarais et al., 2006 [52] reported that kilning at higher temperatures for short periods (5 h at 40 °C, 3 h at 50 °C and 3 h at 60 °C) led to an increase in the activity of the amylolytic enzymes and produced the highest levels of total soluble nitrogen in buckwheat wort when the mashing was carried out in optimal conditions.

De Meo et al., 2011 [107] recommended the use of a NaOH solution in steeping. They found that this had a positive effect on the quality of the malt, increasing both total soluble nitrogen and total amino nitrogen in beer wort, compounds needed to ensure proper fermentation of it.

Zarnkow et al., 2005 [108] used dehulled buckwheat for malting and determined the optimal germination parameters: germination temperature, 19 °C, germination time, five days. They also recommended that the technological operation of kilning the green malt be carried out in several stages so as not to affect the enzymatic activity. Wijngaard et al., 2006 [106] studied the effect of germination time on the quality of buckwheat malt and concluded that at an air temperature of 15 °C for four or five days of germination, buckwheat grains are sufficiently modified, but the nutrients are not yet exhausted. A study by Agu et al., 2012 [104] concluded that high levels of α amylase are recorded in buckwheat malt when the germination temperature was 20 °C.

Phiarais et al., 2005 [52] investigated the impact of the technological operation of kilning green buckwheat malt on the activities of the enzymes α-amylase, β-amylase, β-glucanase, and protease from buckwheat malt-finished product, under optimized steeping and germination conditions. The study found that all these enzymatic activities decrease during kilning at 40 °C for 48 h. They recommended that the technological kilning operation should be carried out in two stages, at different temperatures and for shorter periods. Zweytick et al., 2005 [109] obtained buckwheat malt by applying 2 h of steeping, 4 days of germination, and 26 h of kilning at 80 °C. The beer produced from this malt was opaque, brown in colour, with poor foam stability and bitter taste.

Obtaining 100% buckwheat malt beer was a real challenge for the specialists in the field. The first stage in the production of buckwheat beer that has been optimized is brewing. Buckwheat malt wort had low fermentability values and high viscosity levels compared to barley malt wort [45]. Buckwheat malt has a lower amylolytic activity than barley or wheat malt, which can lead to difficulties in obtaining the finished product beer, such as low extract yields, the high viscosity of beer wort, low rates of filtration, and fermentation problems [26]. Phiarais et al., 2006 [58] showed that the addition of commercial enzymatic preparations improved the filtration process and the quality of buckwheat beer by increasing the extracted content, yield, and total fermentable extract. These effects were achieved by Wijngaard and Arendt, 2006 [110] who developed a mashing diagram for 100% buckwheat malt, by optimizing the two important parameters, temperature and mashing time. Phiaris et al., 2010 [111], obtained a 100% buckwheat malt beer with characteristics similar to a wheat beer, in terms of pH, nitrogen content, degree of fermentation, and alcohol content. In addition, a sensory analysis showed that this beer was acceptable in terms of smell, purity of taste, tingling, and bitterness. In the mashing process it is recommended to observe the following parameters: 15 min at 35 °C or 15 min at 45 °C; 40 min at 65 °C; 30 min at 72 °C; and 10 min at 78 °C. Although the optimum buckwheat gelatinization temperature was found to be 67 °C, the optimum saccharification temperature was 65 °C.

All this research has led to promising results in terms of the quality of 100% buckwheat malt beer; however, studies are needed to optimize the conditions of malting and mashing. Moreover, the quality parameters of buckwheat malt and the sensory characteristics of buckwheat-finished product beer are aspects that have not yet been investigated in depth [26].

#### 2.3.2. Amaranth

There is little information in the literature on the use of amaranth as a raw material in the beer industry, but its use as an unmalted adjuvant can be considered an innovation that will attract consumer interest [25,45].

The first use of amaranth in the production of beer was as unmalted cereal, replacing part of the malt with this pseudocereal. The replacement of part of the malt with unmalted amaranth favourably influenced the amino acid and fatty acid profiles. The type and concentration of amino acids and fatty acids in fermented wort substantially affect the aromatic compounds synthesized by yeast, differences in the profiles of esters and higher alcohols have been reported in beers produced with amaranth [112,113]. The inclusion of amaranth as a non-malted pseudocereal led to a finished product with lower alcohol content and improved antioxidants and polyphenols. Disadvantages include the lower carbohydrate content of amaranth, reduced enzyme capacity, higher gelatinization temperature (72 °C), the need for a specific diagram for mashing, and some small investments to control enzymatic activity [33,113]. To minimize these disadvantages, the mashing process should be modified by applying additional boiling to compensate for the gelatinization temperature and adjusting the pH of the slurry to preserve the enzymatic activity. Pre-treated cereal extracts, hydrolysed starch, or sugar syrup may be added during the boiling of the wort or exogenous enzymes in the mash. The obtained must have high viscosities of 2.0–13.3 mPa s, a pH of 6.2 and a darker colour of 13–14 EBC [114].

Adding a small amount of unmalted amaranth, due to its nutritional and sensory properties, can contribute to the qualities of the finished product, in addition to enriching the beer wort with metal ions essential for the multiplication of yeasts in the fermentation process. According to Cadenas et al., 2021 [25], the use of amaranth as an adjunct in brewing increased the ratio of Mg^2+^/Ca^2+^, necessary for the efficient transformation of fermentable carbohydrates from beer wort into ethyl alcohol, as well as the content of Zn^2+^ and Mg^2+^, even at an amount of 10% amaranth used in the manufacturing recipe.

Amaranth has also been subjected to malting. Due to the small size of the amaranth grains, a steeping time of 1 h was sufficient. The germination operation lasted 3 days, and the kilning was performed at 80 °C for 24 h [33]. Another malting process consisted of steeping at 8 °C, according to the following diagram: 5 h of rest in the water, 8 h of rest in the air, 8 h of rest in the water, 12 h of rest in the air. The germination operation lasted 7 days at 8 °C and 3 days at 15 °C due to insufficient root growth. Kilning was performed at 50 °C for 16 h, at 60 °C for 1 h and 65 °C for 5 h [107].

Zarnkow et al., 2005 [108] used response surface methodology (RSM) to optimize the amaranth malting process to obtain a beer and recommended a steeping time of 36 h to a humidity of 54% and a germination temperature of 8 °C, for 168 h. The malt—as the finished product—had a low content of free amino nitrogen (FAN), and the fermentation performance was poor [108]. The resulting 100% amaranth malt beer was slightly opaque, with poor foam stability and an intense and bitter taste [33]. Research into optimizing the malt quality for beer must be continued in an attempt to raise the diastatic power of the malt of this pseudocereal to values close to the minimum recommended for this purpose, which is 220 WK [119].

Amaranth malt has been used to make gluten-free beer [88,120]. In recent years, the demand for high-quality gluten-free foods, including gluten-free beer, has grown sharply, and the availability of gluten-free, healthy, and tasty beer appreciably improves people’s well-being and perception of normal social life [121].

Table 3 summarizes data from the literature on obtaining beer from amaranth or amaranth malt. Many of these data come from research conducted on pilot scale or in the laboratory on small quantities of raw materials. Technical-scientific progress will have to intervene in the industrial production of beer, by optimizing the technological process and increasing the shelf life of the finished product [8].

### 2.4. Other Uses

Pseudocereals, like cereals, have a high economic value and have multiple directions of use. On the other hand, pseudocereals are important crops for food security in various parts of the globe, such as Asia and Africa. Buckwheat shoots, germs, and micro plants are generally considered to be a rich source of bioactive compounds with valuable nutraceutical properties for the human diet [51,55].

Buckwheat flower is used as an excellent source of honey known for its health benefits due to the presence of benzoic and abscisic acids, flavonoids, and high antioxidant properties [41,52,123]. Buckwheat husks can be used as fertilizer or can be used for packing, filling pillows and mattresses [27,52].

Buckwheat seeds are used as food for birds and animals and in human food [55,82]. Buckwheat is used in the food industry in the form of flour or groats for obtaining bakery products, pastries, and confectionery, biscuits, snacks, pasta, yogurt, being used as a source of protein to products for people suffering from coeliac disease, without gliadin and glutenin (gluten-generating proteins) in its composition [1,27,39,41,81,123]. Moreover, the incidence of people with coeliac disease will certainly increase worldwide in the coming years due to increased awareness of the disease and improved diagnostic procedures [79]. Gluten intolerance can be treated by excluding gluten from the diet, so it is recommended that these patients exclude gluten-containing foods from their diet [78]. In India, buckwheat flour is called “kuttu ka atta” and is consumed by Hindus on certain days of fasting, especially during “Navaratri, Ekadashi, Janamashthami, and Maha-Shivaratri”. In Korea, buckwheat flour is used in the manufacture of various traditional products, such as Makguksu, Naengmyeon, and Mug [47,124]. For example, Makguksu obtained from *Fagopyrum tataricum*, is considered an ideal nutraceutical, very nutritious, with the ability to be easily absorbed into the blood [55]. In other countries, buckwheat is used to make specific products, such as “stove”, “kasha”, “porridge”, “crumpet”, “naengmyeon”, “polenta taragna” and “pizzoccheri” [47].

Many buckwheat-based food and pharmaceutical products have been patented worldwide. For example, more than 210 traditional foods, healthy drinks, and pharmaceutical formulations based on *Fagopyrum tataricum* have been patented in China by the Chinese National Intellectual Property Administration [46]. Of these products, about 28% are in the form of tea and 20% are in the form of beer and wine, with a high content of flavonoids. About 57% of healthy buckwheat-based foods are in capsule form, being the most popular form of product on the market, and 88.6% of them have hypoglycaemic and hypolipidaemic effects, which is in accordance with studies showing that *Fagopyrum tataricum* has the effect of lowering fat and blood sugar [46].

The research was extended to the extraction of valuable compounds from the buckwheat plant, which then received various uses. For example, a protein isolated from buckwheat grains has been processed into bioactive peptides for the development of functional ingredients, “healthy” food, and edible films for food packaging [81]. However, the uniqueness of buckwheat proteins and peptides is largely under-explored [81,124,125].

Amaranth is now widely grown around the world for many reasons of interest, including industrial, medicinal, ornamental, fodder, and nutritional purposes. This pseudocereal is considered a multifunctional plant because it has proven its value in providing cereals and leafy vegetables with high essential nutritional value for animals and human food [126,127,128,129]. The amaranth plant has been rediscovered as having medicinal, nutraceutical, industrial, and ornamental benefits are considered one of the most produced and consumed indigenous vegetables on the African continent in particular, with high nutritional potential, which has not yet been fully explored [35].

After the 1970s, due to its unique properties and versatile use, amaranth came to the attention of specialists in various fields [60,67]. The whole amaranth plant is edible, so amaranth is commonly considered an unconventional food plant [32]. Researchers have stated that amaranth is suitable for the development of foods for consumers with some chronic degenerative diseases and due to its richness in macro and micronutrients, it can be used for the development of new foods [130]. Thus, amaranth grains are used as a starchy raw material for alcohol and alcoholic beverages, for the production of fermented products (for ogi, a traditional product of lactic fermentation of cereal porridge in Africa, or instead of soy in shoyu) [60,131]. Amaranth protein drinks recommended for vegans, coeliac patients and those with lactose intolerance have been developed [65,88].

One of these drinks is Kunu, a non-alcoholic soft drink consumed in Nigeria that was developed by fermenting amaranth grains. Argüelles-Lopez et al., 2018 [132] obtained a drink based on amaranth and chia (*Salvia hispanica*) from germinated and extruded amaranth grains. Germination of amaranth seeds and chia seeds has been shown to have a higher protein content compared to extruded seeds. Steeping and germination are traditional and highly effective treatments for increasing the nutritional and bioactive potential and reducing the anti-nutritional components of pseudocereals [19].

Amaranth grains can be processed into various forms, such as expanded, roasted, flaked, extruded, and ground into flour, and can be used to make bakery products, biscuits, pasta, pastries, confectionery, breakfast cereals, and energizing bars [33,34,92,133]. Amaranth is the most studied pseudocereal for bread making [68]. Kurek and Krzeminska, 2020 [134] suggested that adding 5% amaranth flour was enough to maintain the quality of bread and adding 10–15% could extend the shelf life of finished products. Amaranth has been tested and recognized by many authorities as gluten-free food, suitable for incorporation into the diet of patients with coeliac disease [60].

In Latin America and the Himalayas, amaranth flour is used to produce a variety of flatbreads, as well as in Ethiopia where Kita (unleavened bread) is obtained in various parts of Asia where chapati is prepared [34,63]. Amaranth flour can be used in a mixture of wheat flour to make bakery products. Amaranth grain flour has been shown to contain 50–75% of the flour mixture, retaining its functional properties as well as flavour [59]. Ayo, 2001 replaced wheat flour with amaranth flour (5–50%) and found an increase in water absorption and moisture content of the bread with a decrease in the volume and texture of the finished product [135]. Sanz-Penella et al., 2013 [136] and Miranda-Ramos et al., 2019 [137] found similar results: the specific volume of the bread and the brightness of the crust decreased when the amaranth flour was used in a proportion of more than 25% [86].

In the making of gluten-free bread, amaranth flour was used to increase the protein and fibre content [90,138]. Studies show that adding amaranth to gluten-free bread increases water retention and improves viscoelastic properties and micronutrient content in the finished product [21,139]. Amaranth flour has been used to make nutritious, gluten-free noodles [90].

Young boiled amaranth leaves and shoots can be used as a side dish, in soups, lasagne, pasta, pies, soufflé, etc. [59]. Whole grains are used to make culinary products, such as snacks (alegrias in Mexico; turrones in Peru) obtained by mixing popped seeds with molasses, tasty soups, stews, sauces, porridge, tortillas, pancakes, dumplings, baby purees and soufflé, and boiled grains can be used, such as rice and couscous [34,60,63,70,85,130].

They are also usually picked fresh, used as greens in salads or blanched, steamed, boiled, stir-fried, or baked to taste. In India, amaranth grains are used as a gastronomic substitute for wheat, they have a higher amount of fibre and protein. Amaranth seeds are eaten with boiled rice, used to make “laddoos”, and expanded grains are used to create a confectionery product after mixing with molasses and honey [63]. Amaranth flour can be used as an alternative binder to maize starch in the production of sausages [140]. Amaranth starch can be used as a thickening agent due to its excellent freeze–thaw stability and low amylose content. Amaranth starch is also resistant to degradation, so it can be used in salad dressings, cream soups, and pie fillings [68].

Amaranth grains have been used as a source of protein in feed mixtures, especially after the appearance of bovine spongiform encephalopathy and the consequent ban on meat and bone meal in the diet of all species of farm animals in Europe [60]. The use of grains and/or amaranth leaves and stems in poultry, pig, cattle, sheep, rabbit, and fish feed has shown positive results in terms of productive performance and animal health [32].

The stems of the dried amaranth plant are used as fuel [85]. Non-food applications of amaranth also include starch for laundry, cosmetics, paper coatings, and biodegradable films [63]. Squalene extracted from amaranth grains finds its application in medicine, pharmacy, cosmetology, and even the computer industry [59].

Amaranth has been declared one of the most promising future crops, with a huge potential to feed the global population, a crop that is currently underused with a significant contribution to food and nutrition security [63,64].

## 3. Perspectives

In recent years, the development of beer production from unconventional raw materials through the use of 100% alternative cereals instead of barley or wheat malt has been a major challenge for researchers and specialists in the field [8,141]. In applied research, the use of new raw materials often leads to difficulties in the technological process, such as the need to use exogenous enzymes, excessive saccharification or filtration time, very low wort extract, and therefore low beer alcohol content. It is, therefore, appropriate to extend the research directions in the use of these alternative raw materials, by optimizing the manufacturing recipes and technological processes for the development of new types of beer with improved quality characteristics [142,143,144,145].

Innovation is a specific feature of today’s food industry, and the beer industry is in line with this trend. On the other hand, consumers have begun to pay increasing attention to the supply of products, their nutritional impact, and their beneficial effects on health. Therefore, research must be continued to develop new varieties of beer that respond to these trends [146,147,148,149,150]. The two pseudocereals presented in this study are valuable unconventional raw materials, through the technological, functional, and nutritional properties highlighted in specialized studies and applied research in the field. If buckwheat is about to officially become a raw material that can successfully replace barley malt or wheat malt, the use of amaranth as a 100% malted pseudocereal has led to less promising results. One area of research that has not yet been addressed by specialists is the use of amaranth as a substitute for hops (maybe just hops that are used for bitterness, and not for the flavour) for the bitter taste that beer can give as a finished product.

Future studies are being considered to improve the malting conditions, especially for amaranth, to optimize technological processes and manufacturing recipes to improve the sensory characteristics of beer—the finished product [151,152,153]. This process requires concentrated and continuous research efforts and the coordination of all stakeholders for the effective implementation of relevant solutions with effects on the quality of the finished product [154,155,156]. Technological innovation takes into account technical and economic criteria, but also the acceptability of new assortments by consumers [8].

## 4. Conclusions

Research performed to date on the use of buckwheat and amaranth in brewing has shown the possibility of obtaining new beer varieties, using them from simple adjuvants in the form of unmalted cereals, flour, groats, flakes, 100% extruded or expanded grains buckwheat malt or 100% amaranth malt.

The results indicate that for the further improvement and optimization of the beer quality characteristics as a finished product, combinations of different raw materials may be useful. This may include both buckwheat and amaranth. If buckwheat has so far proved its qualities in obtaining finished products appreciated by consumers, it remains for amaranth to find applicability in this industry, through the functional and nutritional qualities that it possesses, and which have not yet been fully exploited.

In recent years, research studies on the use of buckwheat and amaranth in the manufacture of gluten-free beers have intensified due to the increase in the number of people suffering from coeliac disease. These possible raw materials will continue to attract the attention of consumers with different taste and aroma preferences or consumers with medical conditions. The possibilities are limitless when optimizing the manufacturing recipe in the innovation activity of specialists, which also considers the efficiency and effectiveness of the process of manufacturing new varieties of beer.

## Figures and Tables

**Table 1:** Physico-chemical characteristics of buckwheat, amaranth, and wheat.

Grain	Moisture [%]	Protein [%]	Fat [%]	Carbohydrate [%]	Fibre [%]	References
Wheat	12.8	11.8	2.5	71.2	12.5	[39]
	-	10.91	1.82	75.56	2.2	[64]
	13	14.0	2.0	69.0	1.0	[61]
	-	10.7	2.0	75.4	12.7	[32]
	12.6	11.7	2.0	71.0	2.0	[93]
	-	9–18	2.5–3.3	75–80	2.0–2.5	[94]
	-	8–13	3–4	85	12	[95]
	13	13.7	1.9	72.6	12.2	[96]
	-	11–14.1	1.4–2.1	81.3–83.1	2.1–2.9	[97]
	-	12.9–19.9	1.5–2.0	80	7.7–11.4	[98]
Buckwheat	13.4–19.4	10.4–11.0	2.4–2.8	67.2	8.6	[99]
	-	9.5–14.1	1.8–3.1	80.5–84.1	-	[73]
	-	12–19	1.5–3.7	60–70	1.7–8.5	[55]
	-	10–12.5	4.7	65–75	-	[82]
	11	12	7.4	72.9	17.8	[39]
	-	13.3	3.4	71.5	10.0	[32]
	-	13.9–16.4	3.43–3.86	67.8–78.3	3.55–5.86	[66]
	-	12.28–15.61	1.72–2.24	77.36–81.38	20.32–21.45	[100]
	10.8–11.6	8.51–18.87	1.5–3.7	60–70	2.7–21.3	[101]
	11.2	12.3	2.3	73.3	10.9	[102]
Amaranth	6–9	13–18	6–8	63	4–14	[99]
	-	15.7	7.2	62	4.2	[59]
	-	16	7	62	10	[32]
	-	13.1–21	5.6–10.9	48–69	3.1–5.0	[60]
	6.23–6.71	13.58–17.6	6.3–8.1	58.6–68.9	3.4–5.3	[34]
	-	13.6 ± 0.8	7.3 ± 0.3	69.0 ± 0.2	11.0 ± 0.2	[88]
	11.29	13.56	7.2	65.25	6.7	[64]
	6–9	13–18	6–8	63	4–14	[61]
	-	13.6	7.0	65.3	6.7	[32]
	-	15.1–16.4	6.47–7.25	57.3–65.5	6.53–11.16	[66]

**Table 2:** Published studies on buckwheat malt.

Raw Materials	Technological Process	Study Conclusions	References
Organic buckwheat from the USA	Steeping: 15 °C following this program: 3 h water rest, 3 h air rest, 2 h water rest, 1 h air rest, 1 h water rest, 1 h air rest. Germination:15 °C for 4 days Kilning: 5 h at 40 °C, 3 h at 50 °C and 3 h at 60 °C.	Finished product properties: Moisture = 5.32% pH = 6.05; Extract = 88.7% d.m. Apparent attenuation = 81.0% Free amino nitrogen (FAN) = 231 ± 1 mg/L Total soluble nitrogen (TSN) = 1.300 ± 3 mg/L	[107]
Buckwheat harvested in 2018 in in northwest China	Steeping: 5 h wet stage, 19 h air stage, 4 h wet stage, 20 h air stage Germination: 16 °C for 4 days	Buckwheat malt is a potential material for the beer brewing industry	[103]
Buckwheat from Boston Seeds	Steeping: 20 °C for 20 h, followed by a 4 h air-rest and further 22 h wet-steep. Germination: 20, 25, and 30 °C for 4 and 5 days.	Inclusion of buckwheat as brewing raw materials will increase the availability of suitable materials for use in the production of gluten-free beer, potentially making it more sustainable, cheaper, and more widely available.	[104]
Buckwheat harvested in 2003 in Eastern Europe	Steeping: 7 h resulted in 35% moisture, 13 h in 40% moisture and 80 h in 45% moisture Germination: 15 °C/4 days Kilning: 45 °C/5 h and 50 °C/12 h	The optimum out-of-steep moisture content for buckwheat is between 35% and 40%, which is a compromise between attaining the desired malt quality and minimising malting loss.	[79]
Buckwheat harvested in 2003 in Eastern Europe	Steeping: 3 wet and 3 dry cycles 10 °C for 12 h Germination: 15 °C for 6 days Kilning: 45 °C for 5 h and 50 °C for 17 h	The optimum germination time of buckwheat germinated at an air-on temperature of 15 °C is four or five days. At this time, the grains are sufficiently modified but nutrients have not yet been exhausted.	[44,106]
Buckwheat from Trouw B. V. (Rotterdam, The Netherlands)	Steeping:12 h/10 °C Germination: 96 h/15 °C Kilning: 48 h/40 °C	It was found that malt and wort made from buckwheat kilned at 40 °C for 48 h with optimized steeping and germination conditions, shows potential as a gluten-free brewing ingredient once kilning and mashing procedures are optimized to ensure survival of the enzymes.	[52]
Buckwheat harvested in 2003 in Eastern Europe	Steeping: 12 h at 10 °C Germination: 96 h at 15 °C Kilning: KR1—48 h at 40 °C KR2—5 h at 40 °C and 11 h at 50 °C KR3—5 h at 40 °C, 3 h at 50 °C and 3 h at 60 °C.	Buckwheat malt kilned using KR3 was found to have the highest level of α-amylase, total β-amylase, and protease activity and also produced the highest levels of TSN and FAN when optimally mashed.	[58]
Buckwheat harvested in 2013 in the mountain area of northern Montenegro	Steeping: 10 °C for 12 h in still tap water (control) and still solution of NaOH (0.1, 0.2, and 0.3% [*w/v*]) Germination: 15 °C for 5 days Kilning: 50 °C for 48 h	Steeping in dilute NaOH (0.1, 0.2, and 0.3%) improves the buckwheat malt quality by increasing TSN, FAN, and diastatic power. This method is proposed for the reduction of mold contamination during buckwheat malting.	[115]
Common unhulled buckwheat	Steeping: 96 h degree of steeping 47% Germination: 120 h at 19 °C	For to optimize the malting conditions has been used response surface methodology (RSM).	[45]
Dehulled buckwheat	Steeping: 120 sec per day and 60 sec for half days Germination: 19 °C for 5 days Kilning: 50 °C for 17 h, 1 h at 60 °C and 5 h at 65 °C.	The optimum malting conditions to enrich bioactive polyphenols in dehulled buckwheat	[116]
Common buckwheat	Steeping: 8 h at 20 °C Germination: 96 h at 20 °C Kilning: 22 h at 60 °C and 18 h at 80 °C	The malting process influences the phenolic compound composition and antioxidant activity of buckwheat	[117]
Organic buckwheat	Steeping: 10 h at 30 °C Germination: 40 h at 23 °C Kilning: 10 h at 42 °C	Significantly increased total folate content in buckwheat by 27%.	[118]

**Table 3:** Data from the literature on amaranth beer or amaranth malt beer.

Raw Materials	Technological Process	Finished Product Characteristics	References
70% of barley malt with 30% of amaranth flakes	Mashing: 30 min at 45 °C, 60 min at 62 °C, 30 min at 72 °C, and 10 min at 78 °C Mashing temperature increased by 1 °C per minute, with continuous mixing; Mash filtration; Wort boiling: 60 min with hop pellets 1.5 g/L; Fermentation: at 10 °C for 14 days; Bottling and maturation for 14 days at 1 °C	The addition of amaranth positively influenced the amino acid profiles, a higher content of fatty acids, including long-chain and unsaturated, which resulted in a greater degree of assimilation of these compounds by yeasts.	[112]
60% of barley malt with 40% of amaranth malt	Mashing: 15 min at 50 °C, increase to 65 °C (1 °C/min), 45 min at 65 °C, 30 min at 72 °C and mashing out at 78 °C for 10 min; Mash filtration Wort boiling: 60 min and rested for 20 min Primary fermentation: at 20 °C for 14 days Maturation: 20 days at 1 °C; Refermentation at 23 °C for 1 month	Lower extract yields Lower volumes of the final beers Beer pH = 4.0	[99]
100% amaranth malt	Conventional process of brewing	Extract content of 79.9% very low alcohol beer (0.64%)	[44]
100% amaranth malt	Conventional process of brewing	Slightly opaque, yellow colour. The foam stability—not good The taste—too bitter.	[109]
70% of barley malt with 30% dehulled amaranth seeds, flakes and popping	Conventional process of brewing	use of amaranth increased the ratio of Mg^2+^ to Ca^2+^ as well as the content of both Zn^2+^ and Mg^2+^ in wort substantially	[25,122]
100% amaranth malt	Mashing: double-decoction method: at 50 °C, part of the mash was removed to a boiler and heated for 5 min at 85 °C; the procedure was repeated to obtain a temperature of 71 °C; boiling for 90 min; cooling to 12 °C; pitched with yeast Primary fermentation at 6–12 °C; Secondary fermentation at 4 °C.	Amaranth beer is slightly turbid with a light-yellow colour. The beer tasted too bitter. The foam stability of the beer is poor Beer stability was satisfactory	[20,24]

## Data Availability

Not applicable.
